# Identification of Enhancer RNA CDK6-AS1 as a Potential Novel Prognostic Biomarker in Gastric Cancer

**DOI:** 10.3389/fgene.2022.854211

**Published:** 2022-04-29

**Authors:** Shifeng Yang, Xiaoming Zou, Hao Yang, Jiacheng Li, Ange Zhang, Lisha Zhang, Changjian Li, Lei Zhu, Zhen Ma

**Affiliations:** Department of Gastrointestinal Surgery, The Second Affiliated Hospital of Harbin Medical University, Harbin, China

**Keywords:** CDK6-AS1, CDK6, enhancer RNA, gastric cancer, prognostic biomarker

## Abstract

**Background:** This study aimed to confirm the role of enhancer RNAs (eRNAs) in gastric cancer and their clinical utility.

**Methods:** We used Cox survival and relevance analysis to identify the candidate eRNAs in gastric cancer and performed Gene Ontology and Reactome pathway enrichment to determine the potential functions of eRNAs. Correlation between eRNA, tumor-infiltrating immune cells, and drug sensitivity was then analyzed.

**Results:** *CDK6-AS1*, a long non-coding RNA cyclin-dependent kinase 6, may serve as a poor potential prognostic biomarker candidate in gastric cancer with a positive correlation with its target gene *CDK6*. The low *CDK6-AS1* expression group showed more frequent mutated driver genes than the high expression group. Moreover, *CDK6-AS1* is involved in a key oncogenic pathway of the cell cycle and RNA transcription. *CDK6-AS1* also shows dysregulations and associations with prognosis at the pan-cancer level. This eRNA may also be associated with immune cell infiltration and drug sensitivity.

**Conclusion:** *CDK6-AS1* may be a potential prognostic biomarker and chemotherapeutic drug sensitivity predictor in gastric cancer.

## Introduction

Gastric cancer was the world’s fifth most commonly diagnosed cancer type and the sixth cause of cancer mortality in 2018, responsible for 1,033,701 newly diagnosed cases and 782,685 deaths worldwide (Bray et al., 2018). During the past few decades, gastric cancer has maintained a high case fatality rate of 75% throughout most of the world and is the main contributor to the global disability-adjusted life-year burden (Soerjomataram et al., 2012; Thrift and El-Serag, 2020). Recently, the prognosis of gastric cancer has improved and its treatment technology has significantly improved (The Cancer Genome Atlas Research Network, 2014; Digklia and Wagner, 2016).

The past few decades have witnessed the rapid progress of knowledge about the role noncoding RNAs play in a wide range of cancers (Martens-Uzunova et al., 2014). An increasing number of researchers have paid attention to eRNAs in the mediation of gene transcription (Natoli and Andrau, 2012; Andersson et al., 2014). Enhancer RNAs can independently activate enhancer activity, and cooperate with other transcription-related factors to initiate the formation of the enhancer-promoter loop, thereby activating the expression of downstream genes and pathways (Kaikkonen et al., 2013; Kim and Shiekhattar, 2015). Dysregulation of eRNAs, specifically in the oncogenic signaling pathway, could result in the formation of a wide range of human cancers (Zhang et al., 2019). For instance, Kallikrein-related peptidase 3 eRNA in prostate cancer was found to promote the transcription of the downstream androgen receptor gene and promote cancer cell proliferation (Hsieh et al., 2014). Recent studies have also shown that the dysregulation of some eRNAs could serve as prognostic biomarkers in a range of cancers such as head and neck squamous cell carcinoma and lung and colon adenocarcinoma (Gu et al., 2019; Miracco et al., 2021; Xiao et al., 2021). The predictive factors of gastric cancer have not been fully identified, and the underlying functions associated with the tumor microenvironment (TME) cells and chemosensitivity.

In this study, we aim to identify potential prognostic eRNAs in gastric cancer, specifically focusing on cyclin-dependent kinase 6 (CDK6)-AS1 and its target gene. We performed pathway enrichment analyses to explore the potential function *CDK6-AS1* may have on tumorigenesis. Furthermore, we validated *CDK6-AS1* expression and the overall survival at a pan-cancer level and analyzed the correlation between eRNA, tumor-infiltrating immune cells, and drug sensitivity.

## Materials and Methods

### Data Collection and Processing

Information from 33 datasets was downloaded from the University of California, Santa Cruz (UCSC) Xena The Cancer Genome Atlas (TCGA) hub (https://xena.ucsc.edu) (Goldman et al., 2020). The dataset included 407 gastric cancer tissues, 32 normal tissues, and 9951 other tumors from different types of cancers, and the RNA expression matrix was transformed to log_2_ (FPKM+1). Ensemble transcript IDs were converted to their corresponding GENCODE v19 using the Gene Transfer Format (GTF) annotation files from humans. The enhancer RNAs and target gene information were obtained from the putative literature, which was previously identified by the PreSTIGE method (Corradin et al., 2014).

### Identification of Predictive eRNAs in Gastric Cancer

To avoid bias, patients with a survival time of less than 1 month were excluded. A total of 375 patients passed the quality control and were used in the following analysis as shown in Table 1. The survival-associated eRNAs were screened using the Cox regression model, with age, sex, and tumor stage adjusted as covariates. We set *p* < 0.05 as the significance cut-off value.

**TABLE 1:** The clinical parameters in TCGA gastric cancer cohort.

Covariates	Type	Percent
Gender	Female	134 (35.73%)
	Male	241 (64.27%)
Age	≤60y	121 (32.61%)
	>60y	250 (67.39%)
Grade	G1	10 (2.67%)
	G2	137 (36.53%)
	G3	219 (58.4%)
	Unknown	9 (2.4%)
M_stage	M0	330 (88%)
	M1	25 (6.67%)
	Unknown	20 (5.33%)
N_stage	N0	111 (29.6%)
	N1	97 (25.87%)
	N2	75 (20%)
	N3	74 (19.73%)
	Unknown	18 (4.8%)
T_stage	T1	19 (5.07%)
	T2	80 (21.33%)
	T3	168 (44.8%)
	T4	100 (26.67%)
	Unknown	8 (2.13%)
Clinical Stage	Stage II	111 (29.6%)
	Stage III	150 (40%)
	Stage IV	38 (10.13%)
	Stage I	53 (14.13%)
	Unknown	23 (6.13%)
Race	Asian	74 (19.73%)
	Black or African American	11 (2.93%)
	Native Hawaiian or other Pacific Islander	1 (0.27%)
	Unknown	51 (13.6%)
	White	238 (63.47%)

### Analysis of Significantly Mutated Genes

The R package maftools were used to compare the mutant frequencies of significantly mutated genes between *CDK6-AS1* as high- and low-expression groups. Mutant types including frame shift, deletion, splice site, frameshift insertion, missense mutation, nonsense mutation, multiple hist, and in-frame deletion were considered in the analysis.

### Gene Enrichment Analysis

Gene Ontology (GO) functional analysis was performed using the clusterProfiler package in R software, and a Reactome pathway analysis of eRNA-related coding genes was performed based on co-expression analysis. Specifically, the GO analysis revealed the function of the biology process (BP), cell component (CC), and molecular function (MF). To avoid accumulation of type-I errors, enrichment items meeting the false discovery rate (FDR) < 0.05 were considered significant.

### Validation in TCGA Pan-Cancer Cohort

The expression data of *CDK6-AS1* and its target gene *CDK6* were obtained at the pan-cancer level as previously described, and patients were classified into low- and high-expression groups according to the median value of *CDK6-AS1* expression. The Cox regression method was used to compare the overall survival difference between the two groups. Covariates of sex, age, and tumor stage were adjusted in the Cox model, and Spearman’s coefficient was applied to the correlation analysis.

### Construction and Validation of CDK6-AS1-Related Prognosis

Univariate Cox regression analysis and Kaplan-Meier analysis were used to screen 6 genes co-expressed with CDK6-AS1. Gastric cancer patients in the TCGA data set were randomly divided into the training set and internal test set. The aforementioned six genes were used for the LASSOCox regression analysis. By using the cross-verification error curve, the best tuning parameter λ is selected through the minimum 10-fold cross-verification in the training set. Based on these six genes, a risk-scoring model is established. Risk score = 0.2637*CTHRC1 + 0.0132*PFN2 + 0.1384*PRSS35 + 0.0355*RTN4 - 0.072*SMPD3 + 0.5459*SYCP2L.

GC Patients in the internal test set and an external cohort were divided into a high-risk group and a low-risk group by the optimal cut-off value of the risk score. The overall survival (OS) rates between the high-risk and the low-risk groups were analyzed by the Kaplan–Meier OS analysis. A two-sided log-rank *p* < 0.05 was considered significant. The time-dependent prognostic value of the prognostic signature was evaluated using the R package“survival ROC.” Area under the curve (AUC) values were used to evaluate the time-dependent prognostic values of the prognostic signature. An AUC >0.60 was considered to be acceptable.

### Analysis of Immune Cell Infiltrates

To evaluate the relationship between tumor-infiltrating lymphocytes (TIL) and the expression of *CDK6-AS1* in gastric cancer, we estimated the expressed fraction of TIL cells using the ssGSEA algorithm by comparing the gastric cancer gene expression matrix with those of the signatures from nine reported TIL cell types (Li et al., 2016). The relationship of the proportion matrix for the nine TIL cells with *CDK6-AS1* was calculated by Spearman’s correlation analysis.

### Prediction of Chemosensitivity

The R package pRRophetic (Geeleher et al., 2017), based on the pharmacogenomics database of the Cancer Genome Project (CGP) cell line data and the Cancer Cell Line Encyclopedia (CCLE), was used to predict chemotherapeutic sensitivity for gastric cancer by an estimation of IC50 (the maximal inhibitory concentration). Default settings were used for the prediction model, including “stomach cancer” for reference tissue type and “cvFold = 10” for ridge regression model training.

### Statistical Analysis

R software (Version 3.6.2) was used to perform analyses in this study. The statistical results are expressed as mean ± standard deviation (M ± SD), and the data comparison of the two groups was analyzed with the Wilcoxon rank-sum test. A value of *p* < 0.05 was used to determine the statistical significance.

## Results

### Screening of Key eRNAs in Gastric Cancer

**FIGURE 1:**
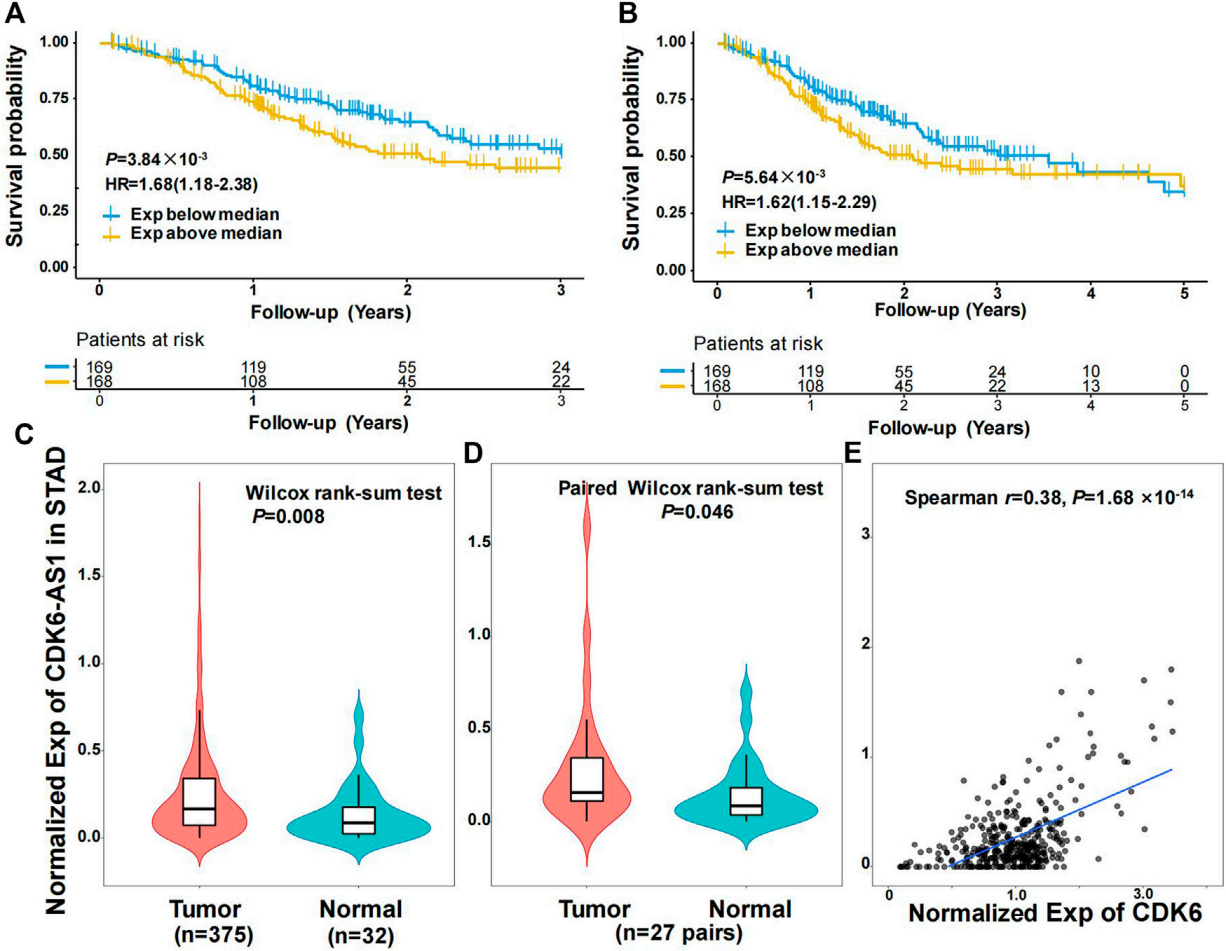
Characteristics of eRNA *CDK6-AS1* in gastric cancer. **(A)** Kaplan–Meier 3-year overall survival curve for gastric cancer patients with *CDK6-AS1* low and high expression. **(B)** Kaplan–Meier 5-year overall survival curve for gastric cancer patients with *CDK6-AS1* low and high expression. **(C)** Differential expression of *CDK6-AS1* between unpaired tumor and adjacent normal tissues. **(D)** Differential expression of *CDK6-AS1* between paired tumor and adjacent normal tissues. **(E)** The correlation between the *CDK6-AS1* and its target gene, *CDK6* expression levels.

**FIGURE 2:**
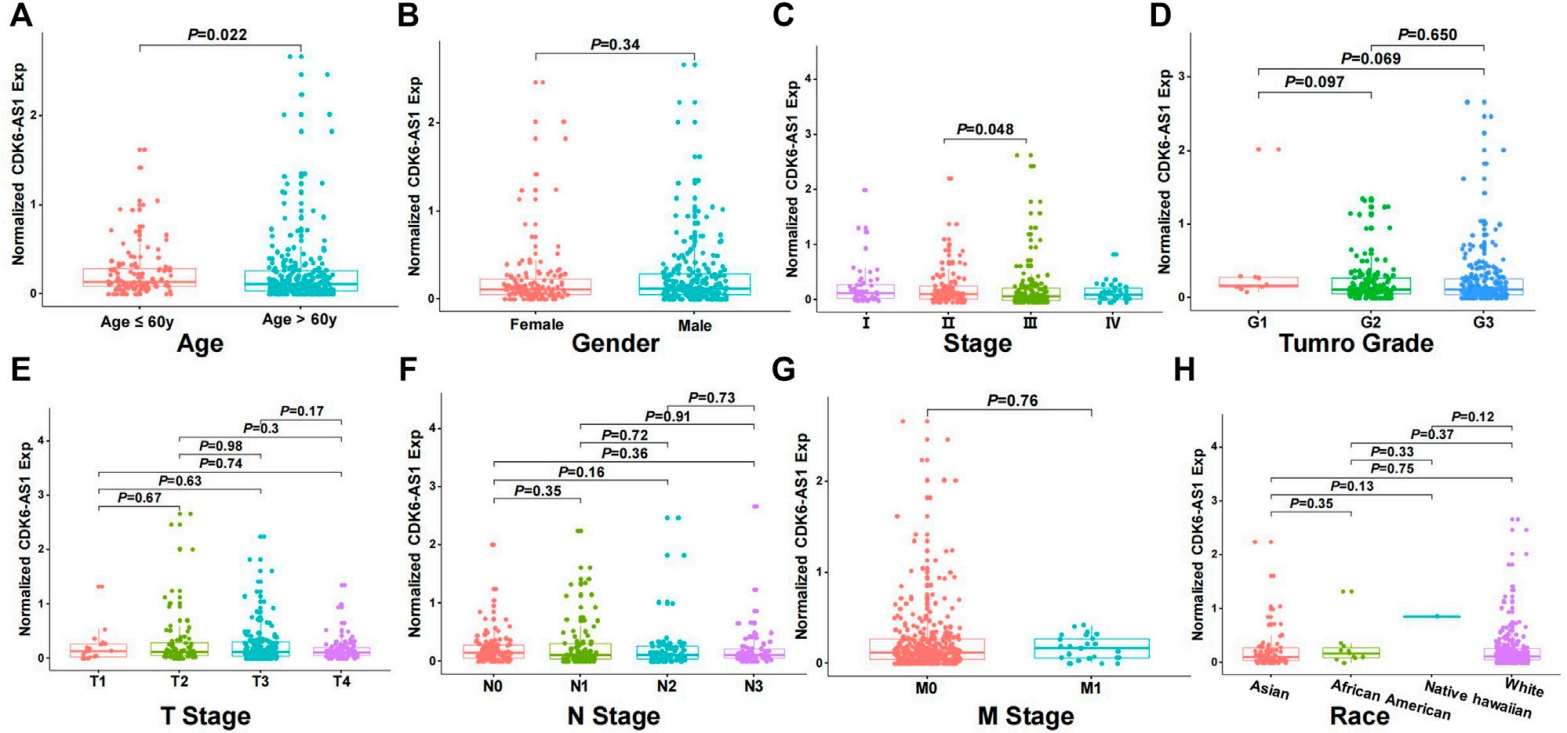
The relationship between *CDK6-AS1* expression and clinical features. **(A–H)** The expression of *CDK6-AS1* among patients with age ≥60 years and <60 years, male and female, I–IV clinical stages, G 1–3 grades, T 1–4 stages, N 0–3 stages, M 0–1 stages, and different races.

**FIGURE 3:**
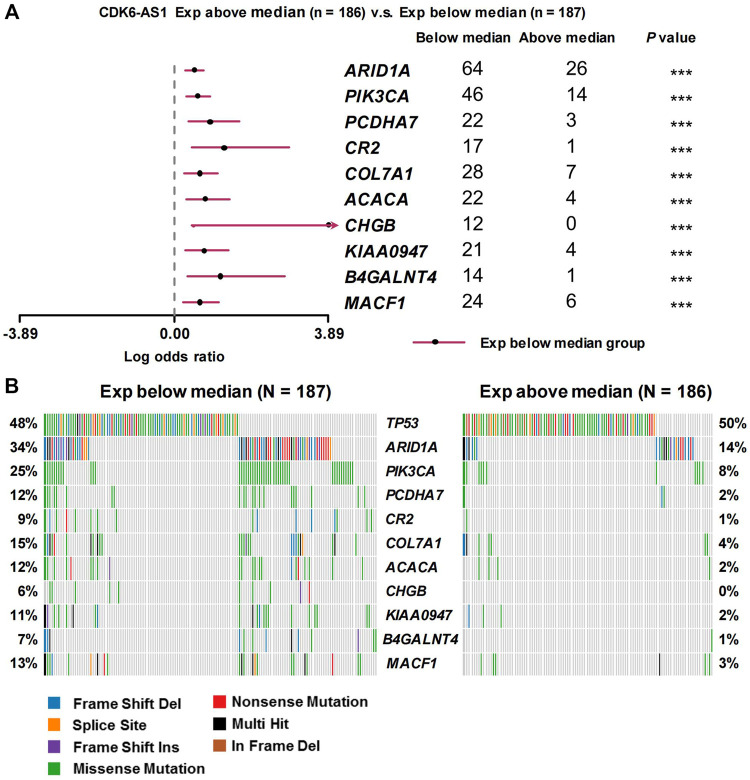
Frequencies of mutated genes between different *CDK6-AS1* expression group. **(A)** Forest plot showing the top frequencies of mutated genes in the high- and low-*CDK6-AS1* expression groups. **(B)** Waterfall plot side by side for comparison of different mutated genes and mutated types between the high- and low-*CDK6-AS1* expression groups.

Twenty-three eRNAs were identified, eight of which met the criteria (Spearman r ≥ 0.3 and *FDR* < 0.05) and were included (Supplementary Table S1). Of these, *CDK6-AS1* exhibited the lowest Cox model *p*-value and was therefore considered a candidate marker. Patients in the *CDK6-AS1* high-expression group had a shorter survival than those in the low-expression group (3-year OS:HR = 1.68, *p* = 3.84 × 10^−3^; 5-year OS:HR = 1.62, *p* = 5.64 × 10^−3^, Figures 1A,B). In addition, *CDK6-AS1* shows a higher expression in unpaired and paired tumor tissues compared to normal tissues (unpaired: *p* = 8.00 × 10^−3^, paired: *p* = 0.046, Figures 1C,D). A positive correlation between *CDK6-AS1* and its target gene CDK6 was observed (Spearman r = 0.38, *p* = 1.68 × 10^−14^). The connections between the clinical features of gastric cancer patients and the *CDK6-AS1* expression were further investigated. It was found that *CDK6-AS1* had a higher expression in patients aged <60 years (*p* = 0.022, Figure 2A). *CDK6-AS1* was significantly linked to the clinical stage (III vs. II, *p* = 0.048, Figure 2C). Other clinical characteristics were not clearly correlated with the *CDK6-AS1* expression (*p* > 0.05, Figures 2B, D–H). As driver gene mutations are crucial to tumor growth, the frequencies of significantly mutated genes were compared between the high- and low-*CDK6-AS1* expression groups. It was noted that several classic gastric cancer driver genes were more frequently mutated in the low-*CDK6-AS1* expression group than in the high-expression group, such as ARID1A and PIK3CA (Figures 3A,B, Supplementary Table S2).

### Pathway Enrichment Analysis of *CDK6-AS1* Co-Expressed Genes

**FIGURE 4:**
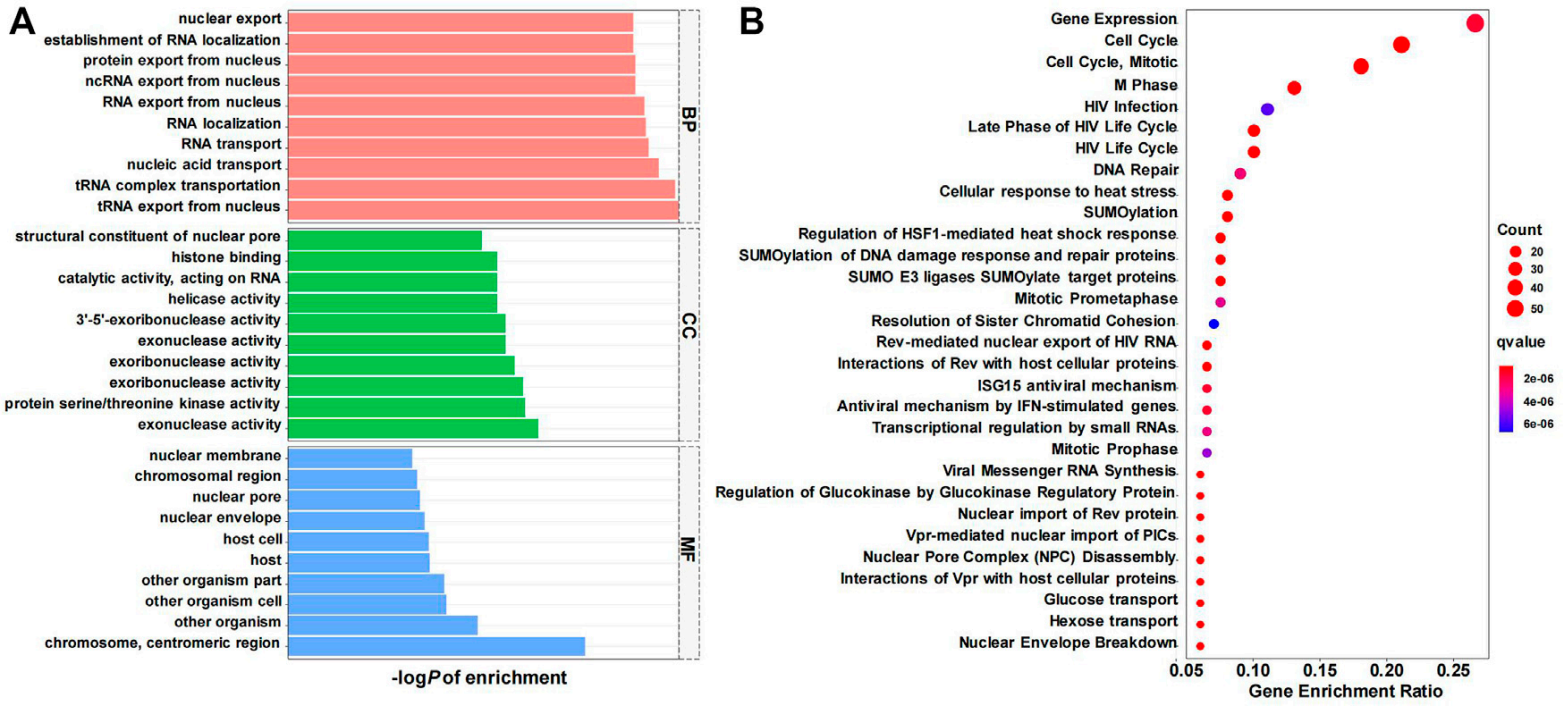
Pathway enrichment analysis of *CDK6-AS1* co-expressed genes. **(A)** GO (Gene Ontology) enrichment result in biological process (BP), cellular component (CC), and molecular function (MF) categories. **(B)** Reactome enrichment result. Top 30 items were shown.

To further explore the function and related pathways *CDK6-AS1* involved in gastric cancer, a co-expression analysis between *CDK6-AS1* and other protein-coding genes in 375 TCGA gastric cancer cases was performed. It was found that 595 transcripts presented a significant correlation with *CDK6-AS1* (Spearman r > 0.30 & FDR <0.05). GO and Reactome enrichment analyses were performed. Results from TOP10GO pathways in BP, MF, and CC are shown in Figure 4A, Supplementary Table S3. In BP, the terms were mainly related to RNA transportation. In MF, the terms were related to exoribonuclease activity. In CC, the term is involved in the nuclear chromosomal region. Enrichment from the Reactome pathway database indicated that *CDK6-AS1* related co-expressed genes were mainly involved in the cell cycle and mitosis (Figure 4B, Supplementary Table S5), and key signals, in tumor cell proliferation, as shown by Spearman’s correlation (r > 0.3 are showed in Supplementary Table S5). Taken together, *CDK6-AS1* and its related genes may be involved in gene transcription and cell cycle processes that are essential for malignant progression.

### Pan-Cancer Analysis of *CDK6-AS1*

**FIGURE 5:**
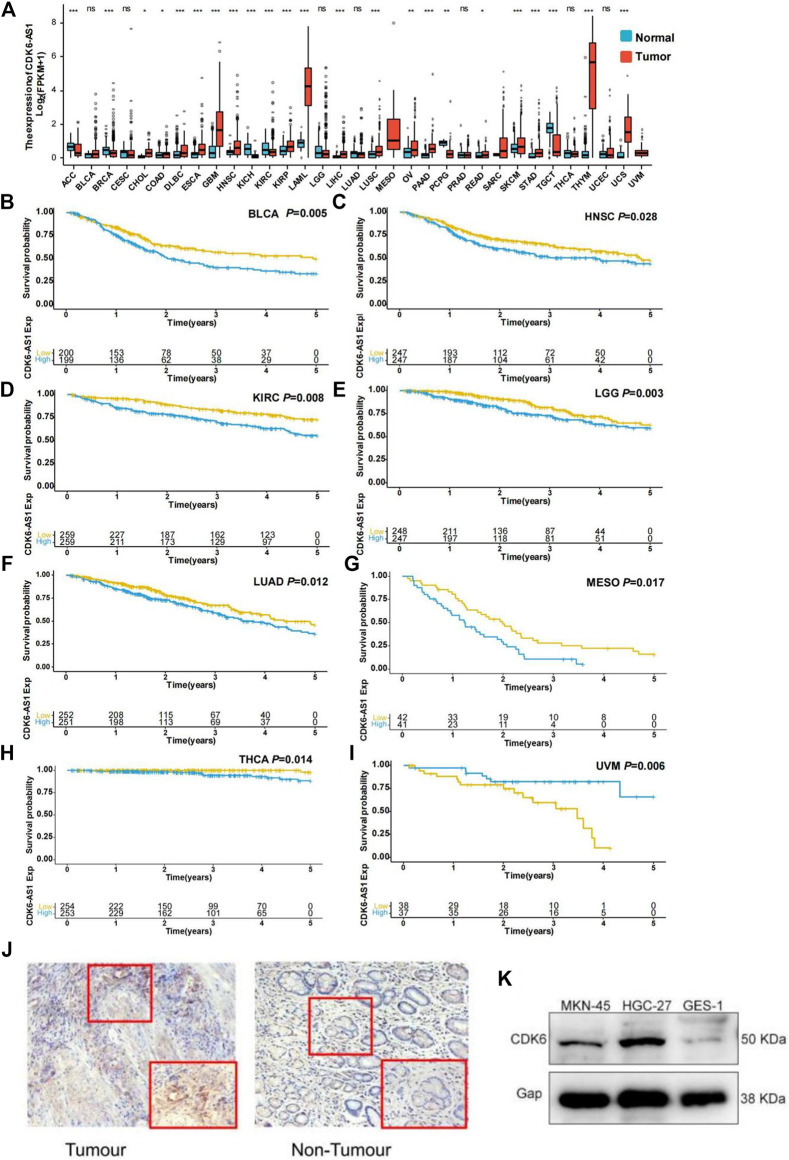
Expression and survival validation in TCGA pan-cancer cohort. **(A)** Differential expression validation of *CDK6-AS1* in TCGA pan-cancer cohort. *p e 0.05; **p < 0.01; ***p < 0.001; ns: not significant with *p* > 0.05. **(B–I)** The prognostic effect of *CDK6-AS1* in other TCGA cancer types. Types with Cox *p* > 0.05 are shown. **(J)** Representative immunohistochemical staining for gastric cancer tumor-infiltrating target gene *CDK6*. **(K)** The *CDK6* was expressed in gastric cancer cells MKN-45, HGC-27, and gastric mucosa epithelial cells GES- 1.

To determine *CDK6-AS1* expression, prognosis, and correlation with its target gene at the pan-cancer level, we analyzed 33 tumor cohorts from the TCGA database. *CDK6-AS1* displayed higher expression in tumor tissues than in adjacent normal tissues in 16 tumor types: COAD, DLBC, ESCA, GBM, HNSC, KIRP, LAML, LIHC, LUSC, OV, PAAD, PCPG, READ, SARC, THYM, and UCS. Meanwhile, six types showed higher expression in normal tissues than in malignant ones: ACC, BRCA, KICH, PCPG, and TGCT (Figure 5A). In terms of survival analysis, high expression of *CDK6-AS1* was related to poor prognosis in BLCA, HNSC, KIRC, LGG, LUAD, MESO, and THCA (Figures 5B–H), while showing better prognosis in UVM (Figure 5I). Further analysis was performed to determine the correlation between *CDK6-AS1* expression and its target gene, *CDK6*. We found that *CDK6-AS1* was correlated with its target in 29 tumor types (Supplementary Table S6). Taken together, when *CDK6-AS1* is dysregulated it could influence the prognosis in a range of different cancer types. Representative immunohistochemical staining was used for gastric cancer tumor-infiltrating target gene *CDK6*. Scale bar, 50 mm (Figure 5J). Western blot results showed that CDK6 was significantly expressed in metastatic gastric cancer cells MKN-45 and in the *in situ* gastric cancer cell line HGC-27, while GES-1 was not significantly expressed in normal gastric mucosa epithelial cells (Figure 5K).

### Construction and Verification of Prognostic Features Related to CDK6-AS1

**FIGURE 6:**
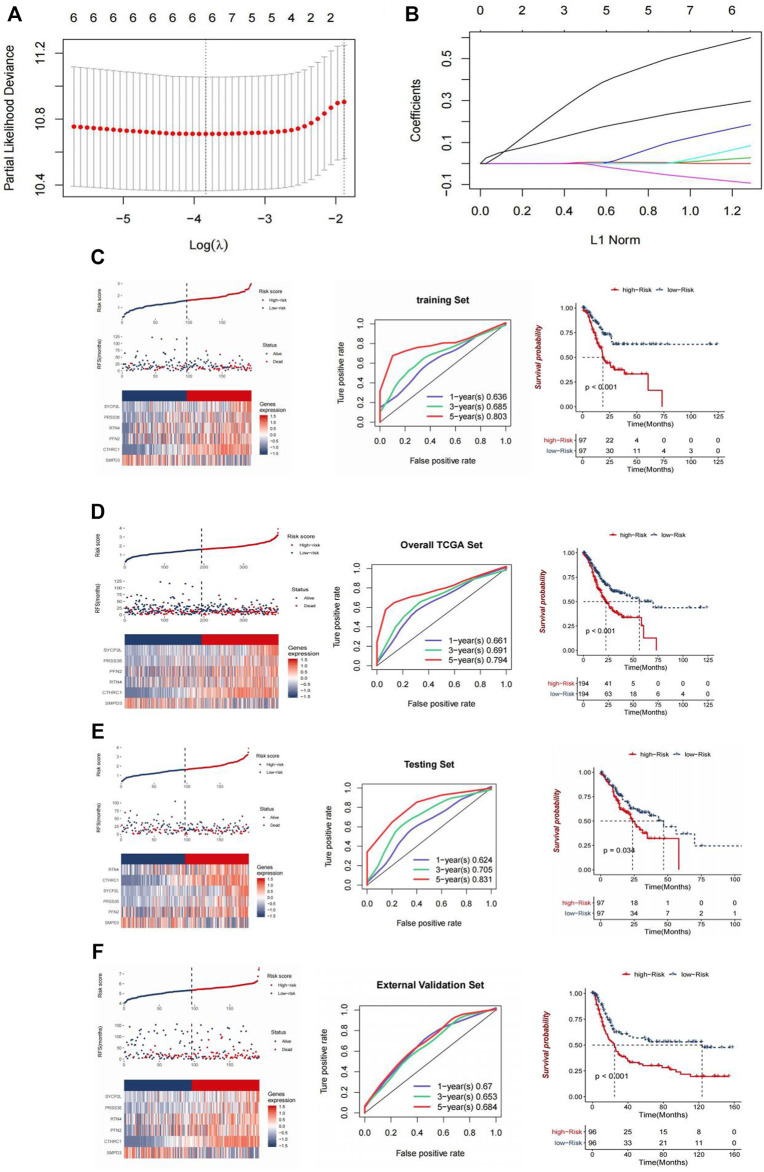
Construction and validation of CDK6-AS1. **(A)** Tuning parameter lambda (λ) selected by cross-validation error curve. **(B)** LASSO coefficient profiles of six genes. **(C,D)** Relationship between the survival status/risk score rank, mRNA expression heat map of 6 genes and survival time (days)/risk score rank. Left: survival status/risk score rank, mRNA expression heat map of 6 genes. Middle: Time-dependent ROC curves for OS of the TCGA-LAML training data set. The AUC was assessed at 1, 3, and 5 years. Right: Kaplan-Meier OS analysis of gastric cancer patients in low-risk and high-risk groups. *p* value was calculated using the log-rank test. *p* < 0.001. **(E,F)** Validation using an internal set **(E)** and an external set **(F)**.

In order to identify predictive genes and construct a prognostic model, six genes related to DEG and OS co-expressed with CDK6-AS1 were crossed to obtain 6 differentially expressed genes related to CDK6-AS1 and OS. Then the six genes were analyzed by LASSOCox regression and the tuning parameter lambda (λ) was selected by using the cross-validation error curve. The prognostic models were constructed when the λ value was minimum (Figure 6A) (Risk score = 0.2637*CTHRC1 + 0.0132*PFN2 + 0.1384*PRSS35 + 0.0355*RTN4 - 0.072*SMPD3 + 0.5459*SYCP2L, and their LASSO coefficient curves are shown in Figure 6B. The relationship between survival status/risk score, mRNA expression heat map of 6 genes, and survival time (days)/risk score showed that the prognostic model had a good prognostic effect, and the OS of gastric cancer patients in the high-risk group was worse than that in the low-risk group (*p* < 0.001) (Figures 6C–F).

### Correlation Between *CDK6-AS1* and TIL

**FIGURE 7:**
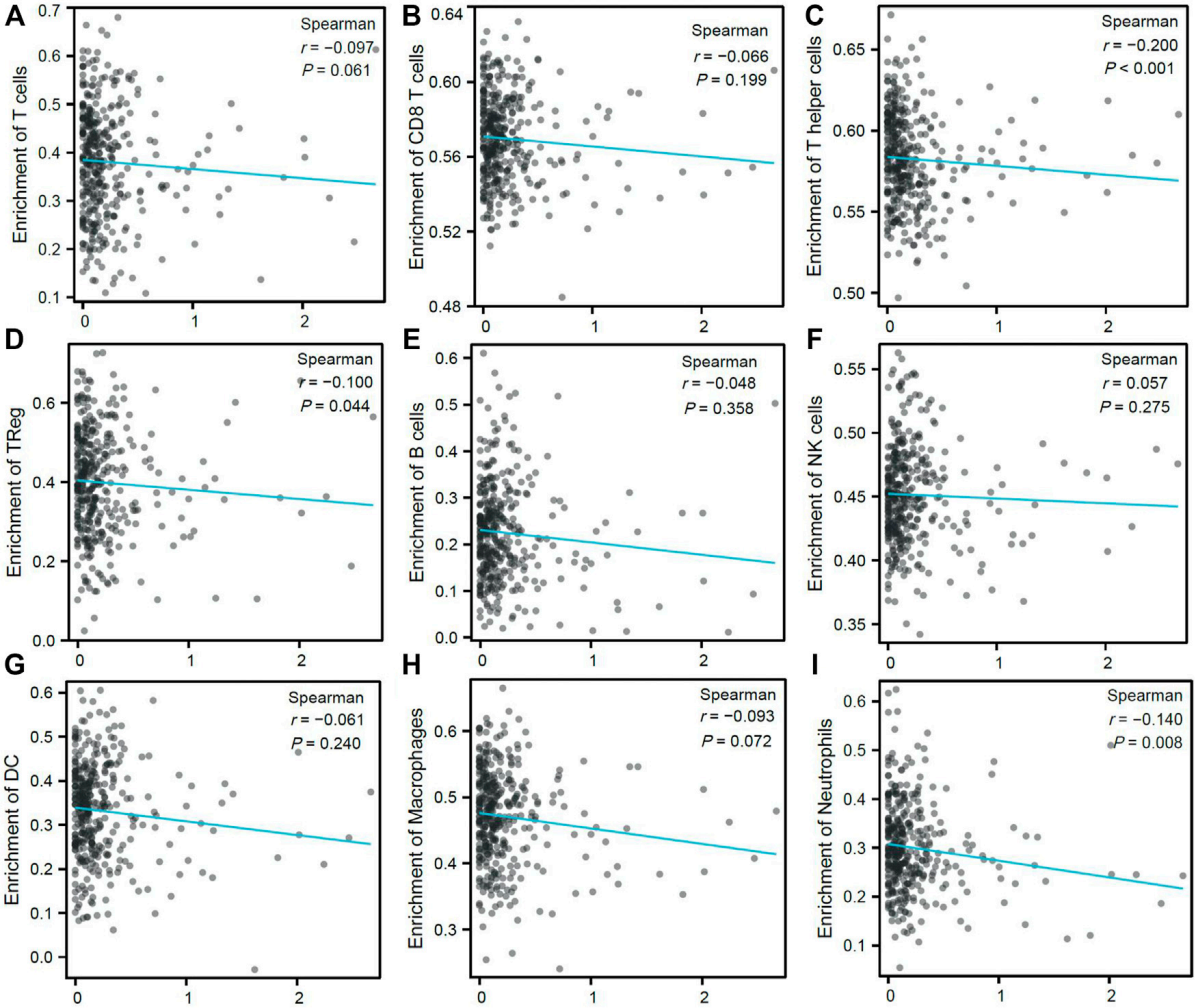
Correlation between expression of *CDK6-AS1* and immune cell infiltration proportion. **(A–I)** Correlation between expression of *CDK6-AS1* and ssGSEA inferred immune cell infiltration of T, CD8+T, T helper, T_reg_, B, NK, DC, macrophage, and neutrophil cells.

TIL are important players in the TME and have been reported to influence gastric cancer survival rates. Therefore, the association between *CDK6-AS1* expression and the proportion of infiltrating lymphocytes was analyzed. Higher *CDK6-AS1* expression showed negative tendencies with almost all inferred immune cell enrichment scores (Figures 7A–I) and was among Th_elper_ (Spearman r = −0.20, e < 0.001, Figure 7C), T_reg_ (Spearman r = −0.10, e = 0.044, Figure 7D), and neutrophil cell clusters (Spearman’s r = −0.14, *p* = 0.008, Figure 7I). Taken together, it appears that high-*CDK6-AS1* expression may hamper immune TME cell infiltration, both in innate and adaptive cell clusters, which may have an effect on anti-tumor immunity.

### Prediction of Drug Sensitivity

**FIGURE 8:**
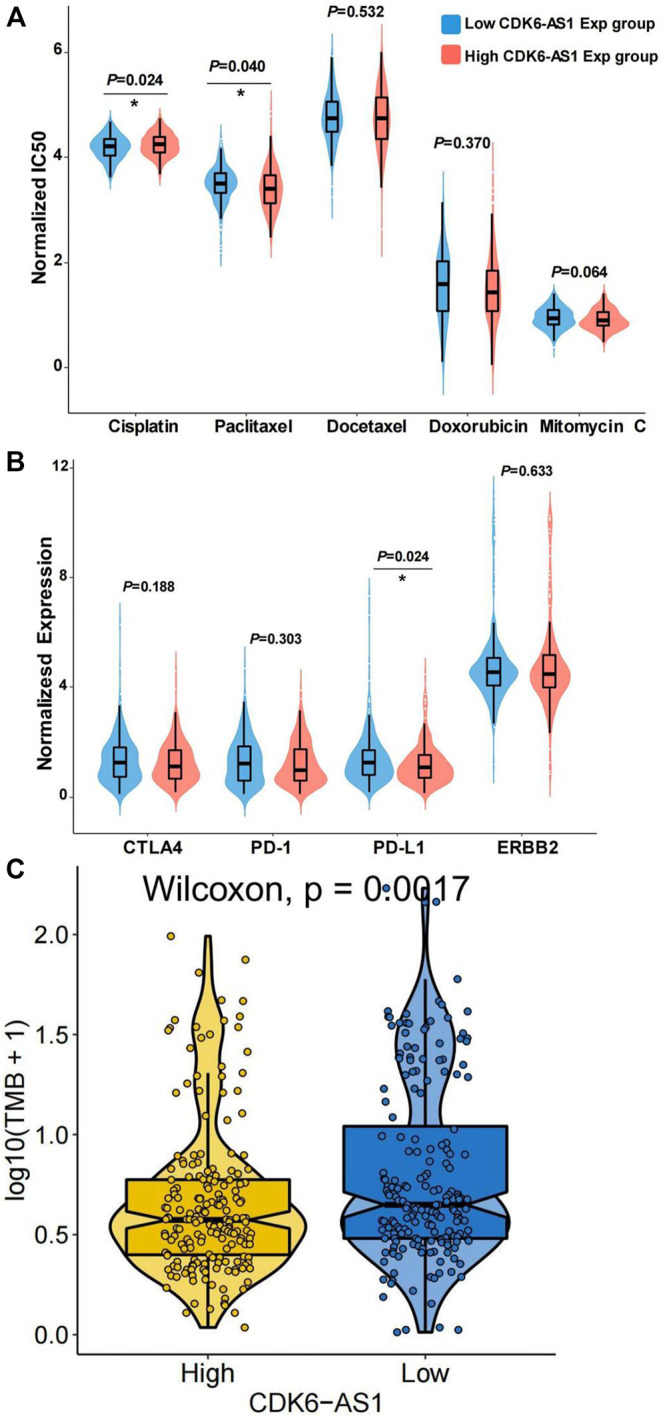
Drug sensitivity prediction in low- and high-*CDK6-AS1* expression group. **(A)** Chemosensitivity prediction of five commonly used drugs in antineoplastic therapy in the low- and high-*CDK6-AS1* expression groups. IC50: Half-maximum inhibitory concentration. **(B)** Expression of immunotherapy and HER2-targeted therapy markers in the low and high *CDK6-AS1* expression groups. **(C)** TMB analysis showed that the immunotherapy effect is better in the group with low expression of *CDK6-AS1*.

Since chemo-sensitivity or -resistance is related to the clinical prognosis of gastric cancer, we explored the chemosensitivity of the high- and low-*CDK6-AS1* expression groups. The ridge regression model was used to predict individual drug sensitivities. A commonly used chemotherapy drug in gastric cancer therapy, cisplatin, showed greater sensitivity in the high-*CDK6-AS1* expression group than in the low-expression group (*p* = 0.024, Figure 8A). Conversely, paclitaxel showed higher sensitivity in the low-expression group (*p* = 0.040). We also explored the expression of immunotherapy and targeted therapeutic markers. PD-L1 expression was higher in the low-*CDK6-AS1* expression group, which may indicate anti-PD-L1 therapy (Figure 8B). We can also prove that CDK6-AS1 is related to immunotherapy by TMB analysis, and the results show that the immunotherapy effect is better in the group with low expression of CDK6-AS1 (Figure 8C).

## Discussion

In our study, we found that *CDK6-AS1* may serve as an independent poor prognostic biomarker candidate in gastric cancer, with a positive correlation to its target gene, *CDK6*. The low-*CDK6-AS1* expression group showed more frequently mutated driver genes than the high-expression group. Moreover, *CDK6-AS1* is involved in key oncogenic pathways such as the cell cycle and RNA transcription. *CDK6-AS1* also shows dysregulation and is associated with prognosis at the pan-cancer level. We also verified by constructing a prognostic model and found that the model had a good prognostic effect, and the OS of patients with gastric cancer in the high-risk group was worse than that in the low-risk group.

Some researchers had demonstrated that eRNAs worked by regulating target genes to form a chromatin loop (Natoli and Andrau, 2012; Bresnick and Johnson, 2019). Research has shown that the eRNA of ACTRT1 can lower the expression of target genes and promote the development of cancer (Bal et al., 2017).

In our study, it was also confirmed that *CDK6-AS1* was associated with survival in eight types of tumors (BLCA, HNSC, KIRC, LGG, LUAD, MESO, THCA, and UVM), and moreover, *CDK6-AS1* expression was correlated with that of its target gene, *CDK6*, in 29 tumor types. Thus, we suggest that *CDK6-AS1* acts as an independent predictor of gastric cancer.

CDK6 can form complex D-type cyclins (D1, D2, and D3) and progresses to the early G1 phase (Nebenfuehr et al., 2020). CDK4/6-cyclin D-complexes are regulated by Cip/Kip proteins, which affect the nuclear translocation of complexes (Song et al., 2020; Nardone et al., 2021). Some studies have reported that CDK6 overexpression could affect lymphoma, leukemia, and other malignancies. Although there is no relationship between mutations in CDK6 and diseases, CDK6 has served as a hub gene in acute myeloid leukemia (Malumbres and Barbacid, 2009; Scheicher et al., 2015). The cyclin D-CDK4/6 axis is commonly expressed in breast cancer. Another effect of CDK4/6 inhibitors is anti-tumor immunity. Zhang et al. suggested that CDK4 negatively regulates programmed cell death ligand 1 (PD-L1) protein stability; moreover, CDK4 and PD-L1 levels negatively correlate with tumor treatment (Scheicher et al., 2015; Goel et al., 2017).

Since TME is important during tumor progress, we studied *CDK6-AS1* expression in infiltrating immune cell fractions. Intriguingly, we found that lower *CDK6-AS1* expression was positively correlated with the proportion of antitumor immune cells, such as T_helper_ cells. This could be partially explained by the finding that the low-*CDK6-AS1* expression group had more mutated genes than the high-expression group. More frequently mutated genes indicate an increased mutational burden and cancer neoantigens (Xu et al., 2020). Neoantigens can serve as targets for immune recognition and recruitment (Garcia-Garijo et al., 2019). The relationship between *CDK6-AS1* and neoantigens warrants further study.

Our study had some limitations. First, the sample size of this research was small, and more clinical research and data are needed. Second, further research and greater detailing is required on the function and role of *CDK6-AS1* in gastric cancer.

## Conclusion

*CDK6-AS1* may serve as a poor independent prognostic biomarker candidate for gastric cancer, demonstrating a positive correlation with its target gene, *CDK6*. Moreover, *CDK6-AS1* is involved in key oncogenic pathways such as the cell cycle and RNA transcription. *CDK6-AS1* also shows dysregulation and is associated with prognosis at the pan-cancer level. These eRNAs may also be associated with immune cell infiltration and drug sensitivity.

## Data Availability

The original contributions presented in the study are included in the article/Supplementary Material, further inquiries can be directed to the corresponding author.
