# Supplementary figures and images for: Identification of Enhancer RNA CDK6-AS1 as a Potential Novel Prognostic Biomarker in Gastric Cancer
Source: Front Genet. 2022 Apr 29;13:854211. doi: 10.3389/fgene.2022.854211 (PMC9100412; doi:10.3389/fgene.2022.854211)

CDK6-AS1

Wilcoxon,  $p = 0.38$

3

2

1

0

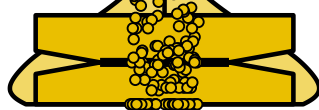

Responder

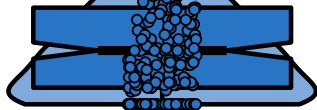

Non-Responder

Immune checkpoint inhibitors

Supplement: Supplementary file 1 [file DataSheet7.PDF]

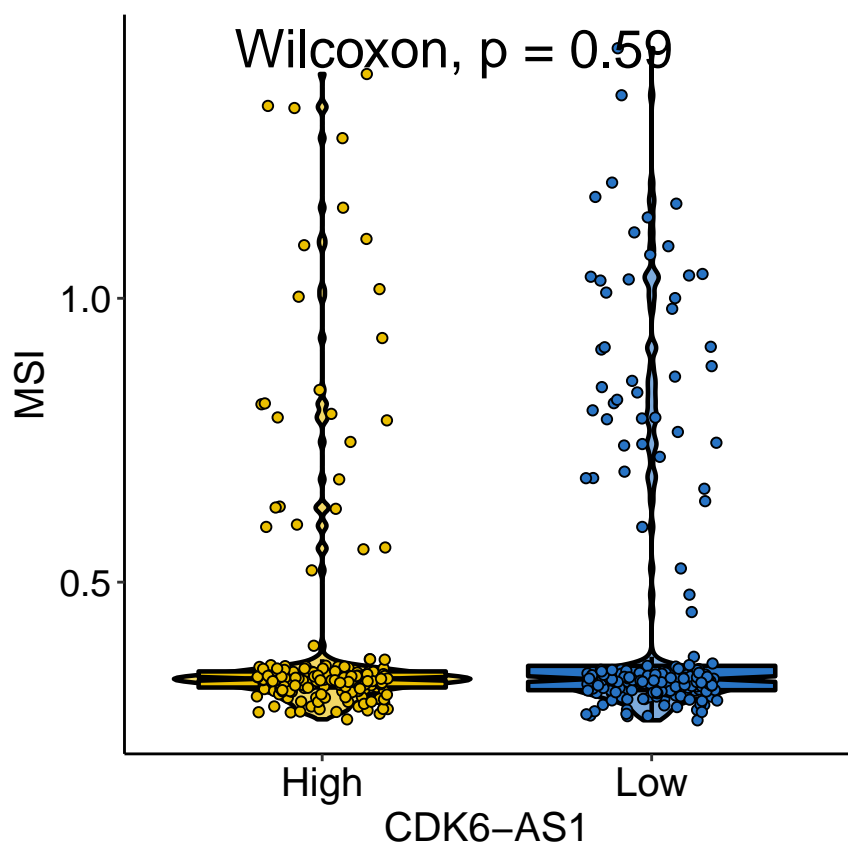

Supplement: Supplementary file 4 [file DataSheet6.PDF]

*P.fisher* =  $4.03e-01$

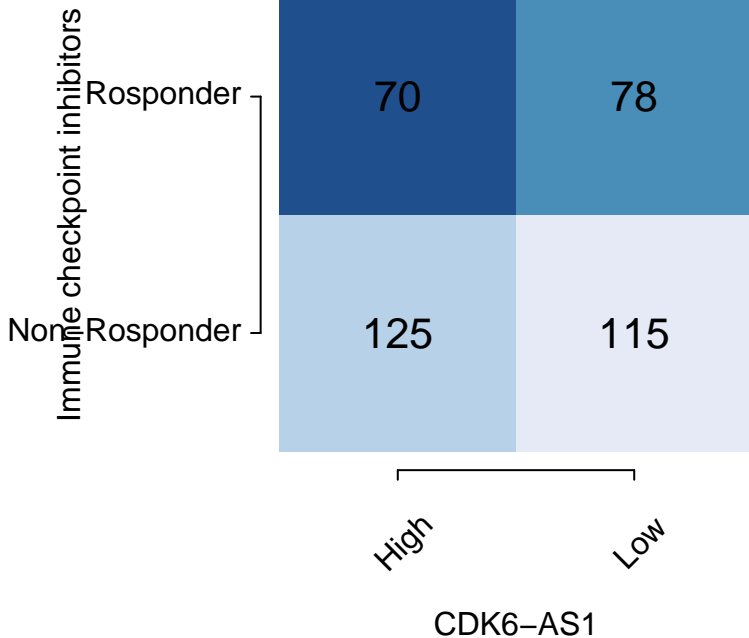

Supplement: Supplementary file 6 [file DataSheet5.PDF]

Wilcoxon,  $p = 0.0017$

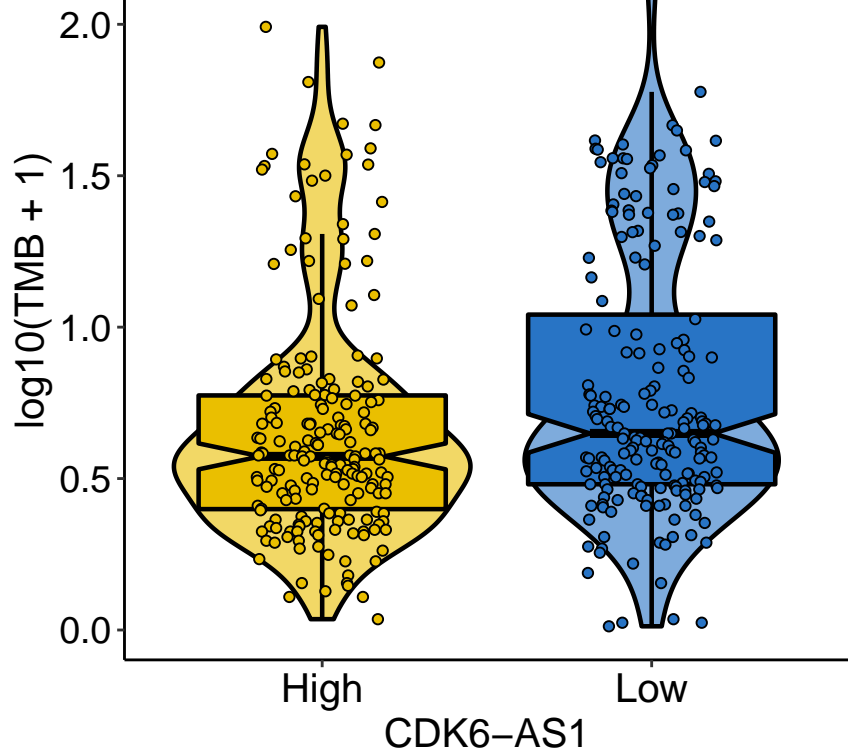

Supplement: Supplementary file 7 [file DataSheet8.PDF]
